# Antimycotic Activity and Genotoxic Evaluation of *Citrus sinensis* and *Citrus latifolia* Essential Oils

**DOI:** 10.1038/srep25371

**Published:** 2016-05-03

**Authors:** Nancy J. Ruiz-Pérez, Marisela González-Ávila, Jaime Sánchez-Navarrete, Julia D. Toscano-Garibay, Mario A. Moreno-Eutimio, Teresa Sandoval-Hernández, Myriam Arriaga-Alba

**Affiliations:** 1 Laboratorio de Investigación microbiológica, Hospital Juárez de México, Av. Instituto Politécnico Nacional # 5160 Col. Magdalena de las Salinas, México D.F. C.P. 07650; 2 Centro de Investigación y Asistencia en Tecnología y Diseño del Estado de Jalisco A.C. Av. Normalistas # 800 Col. Colinas de la Normal, Guadalajara, Jal. México C.P. 44270; 3 Laboratorio de Medicina Regenerativa, Hospital Juárez de México, Av. Instituto Politécnico Nacional # 5160 Col. Magdalena de las Salinas, México D.F. C.P. 07650; 4 Laboratorio de Inmunobiología, Hospital Juárez de México, Av. Instituto Politécnico Nacional # 5160 Col. Magdalena de las Salinas, México D.F. C.P. 07650.

## Abstract

The aim of this study was to evaluate the antifungal activity of essential oils (EOs) of *Citrus sinensis* (*C. sinensis*) and *Citrus latifolia* (*C. latifolia*) against five *Candida* species: *Candida albicans, Candida tropicalis, Candida glabrata, Candida lusitaniae* and *Candida guilliermondii*; and perform its genotoxic evaluation. The EOs of *C. sinensis* and *C. latifolia* were obtained from the peel by hydro-distillation. The major components determined by GC-MS were in *C. sinensis*, d-limonene (96%) and α-myrcene (2.79%); and in *C. latifolia*, d-limonene (51.64%), β-thujene (14.85%), β-pinene (12.79%) and γ-terpinene (12.8%). Antifungal properties were studied by agar diffusion method, where *C. sinensis* presented low activity and *C. latifolia* essential oil was effective to inhibit growing of *C. lusitaniae* and *C. guilliermondii* with IC50 of 6.90 and 2.92 μg respectively. The minimum inhibitory concentrations (MIC) for *C. sinensis* were in a range of 0.42–3.71 μg and for *C. latifolia* of 0.22–1.30 μg. Genotoxic evaluation was done by Ames test where none of the oils induced point mutations. Flow cytometry was used to measure toxicity in human oral epithelial cells, *C. sinensis* was not cytotoxic and *C. latifolia* was toxic at 21.8 μg. These properties might bestow different odontological applications to each essential oil.

Oral candidiasis (OC) is a mucosal illness caused by infection of Candida species, mainly by *Candida albicans* (*C. albicans*)^1^. These infections have been described as a secondary complication in several diseases and it frequently sprouts in immunosuppressed patients that underwent high exposition to antibiotics and corticosteroids. OC is also common on pediatric and elderly patients^2^,^3^,^4^.

The incoming infection with most *Candida* species, other than *C. albicans*, and the growing evidence of antifungal resistance, leads necessarily to seek for new therapeutic alternatives^5^,^6^,^7^,^8^. Throughout human history there has been increasing interest in natural alternative medicine, and nowadays, active principles of medical plants are the focus of scientific papers^9^. In this regard, several studies have shown antioxidant properties derived from flavonoids, carotenoids and vitamin C, which are present at high concentrations in the *Citrus* genus^10^,^11^,^12^,^13^. Essential oils (EOs) from *Citrus* species like *C. sinensis, C. aurantium, C. deliciosa, C. paradise, C. reticulate, C. limon, C. aurantifolia, C. maxima*, have also shown antibiotic and antimycotic activities^14^,^15^,^16^,^17^,^18^,^19^,^20^,^21^. Nonetheless, the biological activities of EOs from *C. sinensis* and *C. latifolia* are still unknown.

The genotoxic effects of many plant extracts and EOs in humans cannot be underestimated, despite its natural origin^22^,^23^,^24^,^25^,^26^,^27^,^28^. Genotoxic evaluation of drugs and any chemical compound or mixtures being considered for clinical applications must be performed accordingly to international institutions such as FDA, IARC and EPA^15^. The Ames test is perhaps the first selected genotoxic evaluation test, designed to detect induced DNA point mutations, it is recommended for its high carcinogenic predictive value and high specificity for detecting mutagenic activities of possible carcinogens in the subsequent rodent trials. Negative results from an Ames test are associated to safety but it is frequently required a second step of validation onto eukaryotic cells system. Additionally, it is highly recommended to have certainty that a new product with potential use in alternative medicine will not be toxic for its target cells^29^,^30^.

The aim of this study was to evaluate EOs from *C. sinensis* and *C. latifolia* as antimycotic on *Candida albicans, Candida tropicalis, Candida glabrata, Candida guilliermondii*, and *Candida lusitaniae* isolated from elderly patients assisting a geriatric clinic of a third-level National Hospital (Hospital Juarez de México). Two additional test of safe usage were performed: Genotoxic evaluation by means of the Ames test and toxicity against oral epithelium cells.

## Material and Methods

### Reagents

EOs of *C. sinensis* and *C. latifolia* were obtained by hydro-distillation of the peel, kindly donated by Frutech International Corporation Cargee Additives, Montemorelos Nuevo León México. Picrolonic Acid (PA), Methyl-N´nitro-N-nitrosoguanidine (MNNG), 2-amino-anthracene (2AA), 4-nitro-quinone-oxide (4NQO) was purchased from Sigma Chemical Co. St. Louis Missouri, USA. Dimethyl sulfoxide (DMSO) was obtained by J.T. Baker Xalostoc, Mexico. Aroclor-1254 was obtained from Supelco Bellefonte, PA; S9 aroclor-1254 induced rat liver homogenate (S9 mix), was prepared as described by Maron and Ames^31^. Amphotericin B was acquired from Laboratorios Pisa S.A. de C.V. México. 7-Aminoactinomycin D (7-AAD) was obtained from Becton Dickinson, Pharmingen^TM^ USA.

### Biological material

*Salmonella typhimurium* strains, TA98, TA100 and TA102, were kindly donated by Dr. Bruce Ames, Berkley University CA, USA. *C. albicans*, *C. tropicalis, C. glabrata, C. guilliermondii*, and *C. lusitaniae*, were previously isolated from the oral cavity of elderly patients from 60 to 104 years old, they were attending to geriatric clinical care (unpublished results).

#### Ethical Considerations

Samples were taken from oral epithelium of elderly patients with clinical data of oral candidiasis. All participants provided informed consent and all experimental methods were carried out in accordance with the approved guidelines. The institutional Comittees of Research, Ethics and Biosafety from Hospital Juárez de México approved the protocol under registration number: HJM2112/12-B and in accordance with “Reglamento de la Ley General de Salud en Materia de Investigación para la Salud” (http://www.conbioetica-mexico.salud.gob.mx/descargas/pdf/normatividad/normatinacional/10._NAL._Reglamento_de_Investigacion.pdf).

### GC-MS analysis

Gas chromatography coupled to a JEOL GCmate mass spectrometer operating in electron ionization (EI) mode were used for component analyses. Mass spectra were acquired scanning from m/z 20 to m/z 250. The constituents of the oils were identified by using standard reference compounds and also by matching the mass spectra fragmentation pattern with NIST Mass Spectra Library in the GC-MS database.

### Antimycotic activity

Antimycotic activities of *C. sinensis* and *C. latifolia* EOs were evaluated against previously isolated strains from patients: *C. albicans*, *C. tropicalis, C. glabrata, C. lusitaniae* and *C. guilliermondii*. Antimycotic studies were done with the agar diffusion method; 20 μl of McFarland 0.5 dilutions (equivalent to 6 × 10^4^ CFU)^32^ were prepared on sterile isotonic saline, from each evaluated Candida. These solutions were inoculated on Sabouraud agar on sterile glass Petri dishes. Then agar was perforated with a 5 mm sterile penicylinders and 50 μl of the EO containing the concentrations described in Table 1. Sterile saline and tween 20% solutions were used as negative controls, amphotericin B was evaluated as a positive control (0.16 to 80 mg/hole) accordingly to M27-A3 document from the Clinical Laboratory Standard Institute (CLSI)^33^. Petri dishes were incubated at 37 °C for 24 hours and inhibition zones were measured. Data were analysed by one-tailed univariant Bonferroni test using SPSS v10 software. Logarithmic regression was performed to calculate values of IC50 and Minimum Inhibitory Concentrations (MICs).

### Genotoxic evaluation

#### Mutagenicity assays

In order to evaluate the bactericide properties of both EOs, 100 μl of overnight cultures of *S. typhimurium* (accordingly to Maron and Ames)^31^ were exposed to several dilutions (Table 2). Strains were diluted to 10^−5^ on sterile saline solution and poured on nutrient agar plates. Plates with no treatment were considered as 100% survival rate. Cultures with survival higher to 80% were chosen for Ames method testing.

Mutagenicity evaluation was done accordingly to Maron and Ames^31^. Positive controls were 2AA (10 μg/Petri dish) with S9 mix for TA98, TA100 or TA102 strains. Positive controls without S9 mix were PA (50 μg/Petri dish) for TA98, MNNG (10 μg/Petri dish) for TA100 or 4NQO (10 μg/Petri dish) for TA102 strains. EO concentrations presented on Table 2 were evaluated in each strain either with or without S9 mix. Treated cells were poured on Vogel Bonner medium plates, incubated for 48 hrs, and then were counted on a semi-automatic fisher colony counter. Results were considered positive when the number of histidine+ revertants was twice the obtained on spontaneous reversion plate.

#### Cytotoxicity assays

Cytotoxicity assays were done using human epithelial cells obtained after scrubbing with interdental brushes the internal oral mucosa of both right and left cheeks from healthy non-smoker volunteers, as described^34^ and samples were transported on 1X PBS. From an original cellular suspension, seven aliquots of 100 μl were taken and separated on sterile tubes; first tube was the untreated control, other tubes were treated with 10 μl of each concentration described on Table 1. 10 μl of H_2_0_2_ were added to the last tube as a positive control. All tubes were incubated for 1 minute and reactions were stopped adding 1 ml of PBS 1X. Samples were centrifuged at 3000 rpm on a Clay-Adams clinical centrifuge (Dynamic) for five minutes, and two washing steps were done with PBS (1X) solution to eliminate residual EO. Viability was measured by flow cytometry analysing stained epithelial cells on a BD Accury^TM^ C6 flow cytometer system (Becton Dickinson, San José CA, USA) with 7-Actinomicine-D (7-AAD Staining Solution, BD Pharmingen™, San Jose California USA, Catalogue number 555816), a fluorescent molecule that is internalized exclusively through damaged membranes after a necrotic process, as cell toxicity indicator.

## Results

### Essential Oils composition

**Figure 1:**
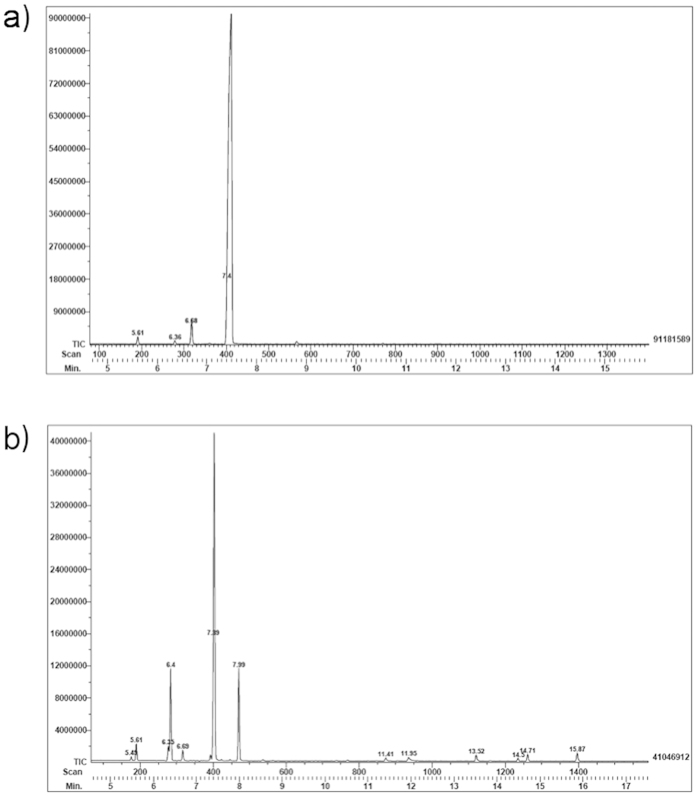
Gas chromatography of the essential oils of (**a**) *Citrus Sinensis* and (**b**) *Citrus latifolia*.

Qualitative and quantitative analysis for the composition of each essential oil are shown on Fig. 1 and Table 3. The chromatograms render different compositions for each EO (Fig. 1), being *C. sinensis* less complex than *C. latifolia*, although limonene was the major component; myrcene and α-/β-pinene were also common components on both oils. In summary, four constituents were detected in *C. sinensis*, d-limonene (96%) and α-myrcene (2.79%) as predominant. Thirteen components were identified on *C. latifolia* and the main were d-limonene (51.64%), β-thujene (14.85%), β-pinene (12.79%) and γ-terpinene (12.8%).

These results showed that there are similarities between both EOs that might contribute to the antifungal capacity, although the amounts of the corresponding compounds are very different.

### Antimycotic activity

**Figure 2:**
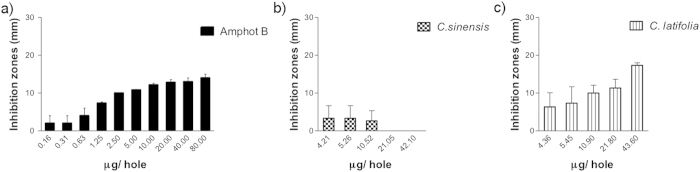
Antimycotic activity of EO of *Citrus sinensis* and *Citrus latifolia* against *Candida albicans*.

Results of EOs antimycotic activity against Candida species are presented on Fig. 2 through 6. Figure 2 show that inhibition halos of *Citrus sinensis* and *Citrus latifolia* on *C. albicans* cultures were statistically different (p < 0.0001) compared to control group. Calculated inhibition halos at 50% of growth were 5.51 and 9.46 mm, respectively. Both presented lower values as compared to amphotericin B (12.05 mm), meaning that growth inhibition was less evident.

**Figure 3:**
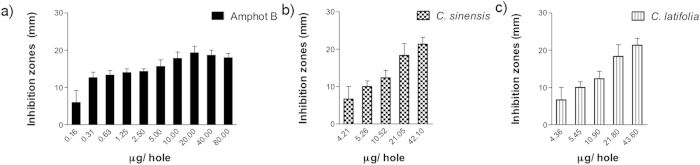
Antimycotic activity of EO of *Citrus sinensis* and *Citrus latifolia* against *Candida tropicalis*.

Measured inhibition halos of *Citrus sinensis* (p = 0.0001) and *C. latifolia* (p = 0.047) on *C. tropicalis* cultures were statistically different compared to control group as it can be seen in Fig. 3. Calculated inhibition halos at 50% of growth were 4.44 and 10.87 mm, respectively. Growth inhibition was less apparent in *C. sinensis* meanwhile *C. latifolia* was similar to amphotericin B.

**Figure 4:**
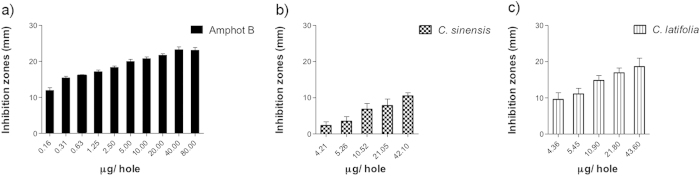
Antimycotic activity of EO of *C. sinensis* and *C. latifolia* against *Candida glabrata.*

Statistically significant differences were found for *Citrus sinensis* and *C. latifolia* on *C. glabrata* cultures (p = 0.000). Inhibition halos at 50% of growth were 5.78 and 8.52 mm, respectively. This indicates that growth inhibition was less than amphotericin B, although it was clearly perceptible (Fig. 4).

**Figure 5:**
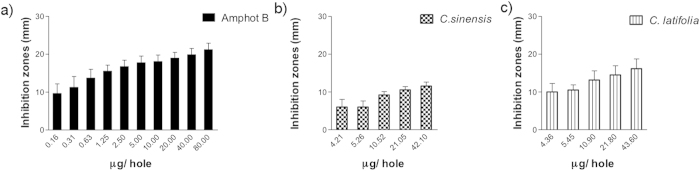
Antimycotic activity of EO of *C. sinensis* and *C. latifolia* against *C. lusitaniae.*

Figure 5 represents the results obtained over *C. lusitaniae* cultures. For *C. sinensis* there was no growth inhibition observed (inhibition halo at 50% of 2.00 mm), with p < 0.0001 when compared with the positive control. *Citrus latifolia*, presented better antimycotic activity (p = 0.807, 8.06 mm) although lower to that obtained with amphotericin B (11.49 mm).

**Figure 6:**
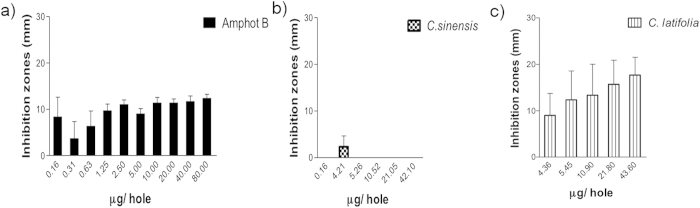
Antimycotic activity of EO of *C. sinensis* and *C. latifolia* against *C. guilliermondii.*

There were no inhibition halos detected on *Candida guilliermondii* cultures treated with *C. sinensis*. On the other hand, *Citrus latifolia* was able to diminish the growth with inhibition halos at 50% of 8.94 mm, significantly higher (p = 0.689) that amphotericin B (6.37 mm) as it can be seen on Fig. 6.

Finally, IC50 and MIC are summarized in Table 4, where *C. sinensis* had its best activity against *C. glabrata* and it has a nule effect with *C. guilliermondii*. Meanwhile C latifolia had effect on all species being the strongest with *C. guilliermondii*, surpassing the effect of amphotericin B.

### Genotoxicity

#### Mutagenic evaluation

**Figure 7:**
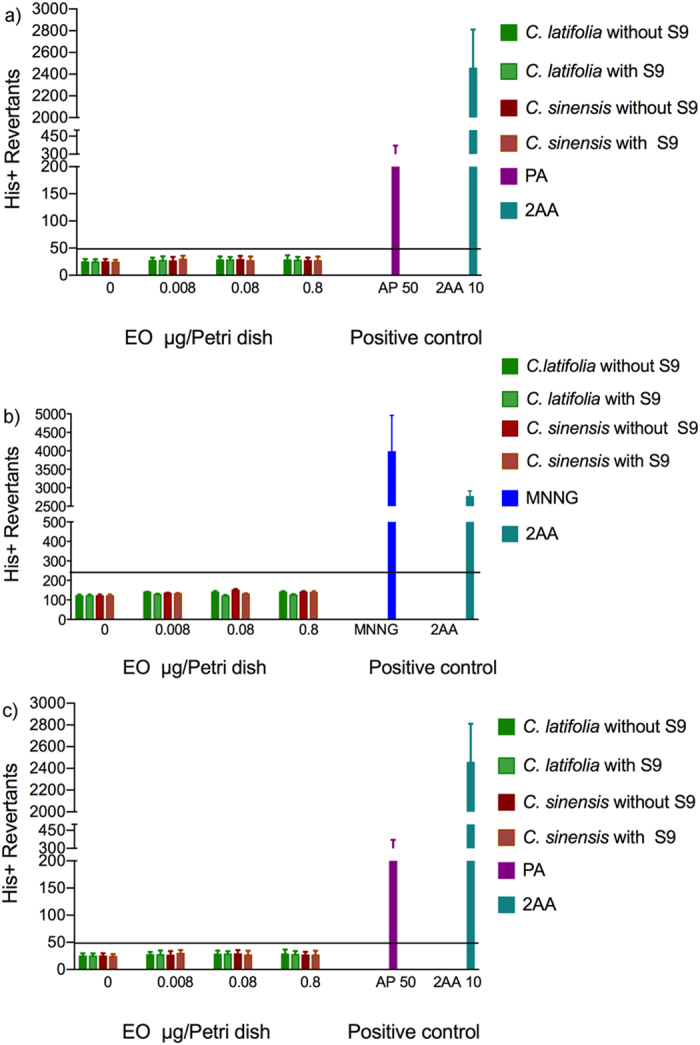
Mutagenic evaluation of EOs. (**a**) Ames test on *Salmonella typhimurium* strain TA98. Spontaneous reversion: 24.67 + 1.7; 2AA: 2-amino-antraceno; PA: picrolonic acid. (**b**) Ames test on *Salmonella typhimurium* strain TA100. Spontaneous reversion: 121.44 + 8.32; 2AA: 2-amino-anthracene; MNNG: metil-N-nitro-N-nitrosoguanidine. (**c**) Ames test on *Salmonella typhimurium* strain TA102. Spontaneous reversion: *305.33* + *57.12;* 2AA: 2-amino-anthracene; 4NQO: 4-nitro-quinone-oxide.

Neither *Citrus sinensis* nor *Citrus latifolia* induced point mutations in the presence or absence of S9 mix. Figure 7a shows that frameshift mutations were not induced on *Salmonella typhimurium* TA98 strain. Results were considered positive when the number of colonies was above the cutting line, as it can be observed for PA and 2AA. Figure 7b show that none of the EOs induced base pair substitution mutations, as it can be observed with the positive controls MNNG or 2AA. These EOs did not induced ROS caused mutations on the *S. typhimurium* TA102 strain. Only positive controls 2AA or 4NQO were able to induce a positive response as it is shown in Fig. 7c.

#### Cytotoxicity

**Figure 8:**
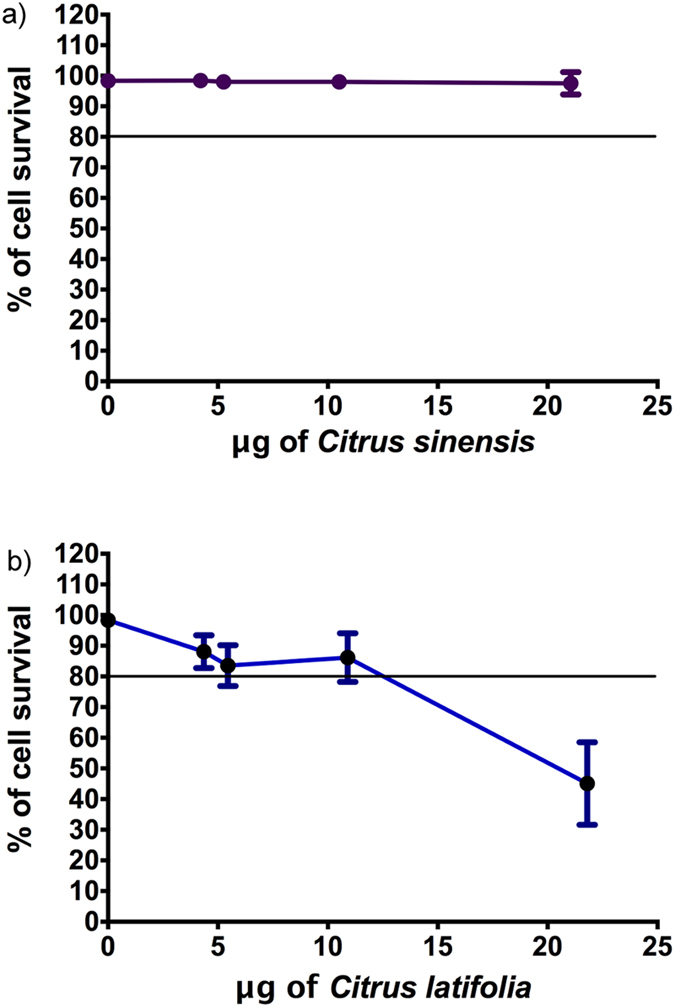
Viability of human epithelial cells. (**a**) After treatment with EO of *Citrus sinensis.* (**b**) After treatment with EO of *Citrus latifolia.*

Figure 8a shows that *Citrus sinensis* had no cytotoxic effect for human epithelial cells at the same doses used for antimycotic tests (see material and methods) since viability levels were maintained over 80% at all probed concentrations. It also can be seen on Fig. 8b that only the highest dose tested (21.8 μg) of *Citrus latifolia* EO was cytotoxic for these cells.

## Discussion

The results presented in this work of antimycotic properties of *C. sinensis* and *C. latifolia* were performed against *Candida* species isolated from clinical cases of elder patients with clinical data of oral candiasis, by contrast to other reports that evaluate of other *Citrus* derivatives on *Candida albicans* and bacterial strains from ATCC^13^,^14^,^15^,^17^,^19^,^20^,^21^. The antimycotic evaluation of the EO of *Citrus sinensis* by agar diffusion method shows inhibitory effect against the studied *Candida* species except on *C. guilliermondii*. Its inhibitory effect was higher for *C. glabrata* (Fig. 3). Nevertheless it was significantly lower when compared with the positive control of amphotericin B. It was noticeable that the background growth in Petri dishes of *C. glabrata* cultures treated with *C. sinensis* was nonconfluent, this is to say, a diminished background was present despite they were plated from the same original 0.5 McFarland suspension, (data not shown). These data reinforce the observation of the antimycotic effect against *Candida spp.*, specifically on *C. glabrata*. On the other side, antimycotic activity was demonstrated for *C. latifolia* on all species, being the highest against *C. guilliermondii*, in this case even better than amphotericin B. It is worth saying, that the antimycotic activity of these EOs is relevant since it was compared to a pure salt such as amphotericin B, a highly potent antimycotic^35^, meanwhile each oil is a mixture of several compounds, each component might contribute to the activity of the assembly, probably with less intensity than if they acted alone; or else, enhance/inhibit individual effects. The analysis of the individual components could be made in the future, as it was beyond the scope of this paper.

However, it could be argued, the chemical compounds like α-pinene, that have antifungal and antibacterial activities^36^, may contribute to the observed activity since is found in both *C. sinensis* and *C. latifolia* oils. Additionally, it has been shown that citral is an agent able to form complexes that interfere with the electron flux in mycotic cells^37^, which could be relevant for the inhibitory activity of *C. latifolia*. On the contrary, it has been demonstrated that limonene, β-pinene and myrcene are implicated on growth stimulation of other species of fungi^38^, which might explain the low activity observed for *C. sinensis*. The analysis of the components on each essential oil revealed the presence of common terpenes to the Citrus taxa^39^. Only one component (*3,7-Nonadien-2-one,8-methyl-,(E)*-), was unusual and it was present in perceptible amounts (0.93%) on the essential oil of *C. latifolia*, and it could be further considered.

Our study presents the mutagenic evaluation of these EOs by means of Ames test, recommended as first strategy in risk-benefit evaluation of new products with possibilities to be used in humans^29^.

In fact, there are not previous mutagenic evaluations of *Citrus* derivatives in current literature, albeit Hammer *et al.* recommended that it should be done^15^. None of the evaluated EOs produced frameshift mutation, base-pair substitution or generates ROS damage when evaluated in the Ames test.

But as it has been formerly commented, not all natural occurring products and plants used in alternative medicine are innocuous. In fact it has been reported that there are some plants having mutagenic, toxic and cytotoxic effects both *in vitro* and *in vivo*, for instance, *Chrysobalanus icaco*^22^, *Urtica dioica* and *Euphorbia rigida*^23^, *Tinctura Alchemillae*, *Cratagei extractum*^25^, *L. stoechas*^26^, *Myrciaria tenella*, *Smilax campestris*, *Tripodanthus acutifolius* and *Cassia corymbosa*, among others^27^,^28^.

It is well known that some of the main components of EOs are vitamin C, flavonoids and beta-carotenes^10^,^12^,^13^ that may have antimutagenic properties, so it will be advisable to further evaluate the antimutagenic properties attributable to these additional components. The cytotoxic effect of *C. latifolia* EO should be evaluated against cancer human cell lines in order to know their possibilities as anticarcinogen.

In the present study *C. sinensis* was not cytotoxic for human oral epithelium cells even at higher doses probed *in vitro*. However, there was a toxic effect observed at the highest dose probed of *C. latifolia* EO (21.8 μg). Although in order to conclude, it is recommendable to perform studies on animal models where other toxicity parameters could be measured.

Consecutively *Citrus sinensis* EO could be used as an ingredient in oral hygiene products as prophylactic agent because it seems not mutagenic, not cytotoxic and it has moderated antimycotic activity. In case of *C. latifolia* it should be employed for products for external use such as elder dental prostheses and orthodontic appliances due to its toxicity. However, these oils could be a less toxic alternative to amphotericin B^35^, although more studies are necessary.

## Conclusions

In conclusion, *Citrus sinensis* has antimycotic activity against *C. glabrata*, it was nor cytotoxic neither mutagenic.

*Citrus latifolia* EO has antimycotic effect against *C. guilliermondii*, it was not mutagenic and doses below at 20μg were not cytotoxic.

## Additional Information

**How to cite this article**: Ruiz-Pérez, N. J. *et al.* Antimycotic Activity and Genotoxic Evaluation of *Citrus Sinensis* and *Citrus Latifolia* Essential Oils. *Sci. Rep.* **6**, 25371; doi: 10.1038/srep25371 (2016).

## Figures and Tables

**Table 1:** Doses employed for antimycotic and cytotoxicity assays.

*Citrus sinensis* (μg/50 μl)	*Citrus latifolia* (μg/50 μl)
4.21	4.36
5.26	5.45
10.52	10.9
21.05	21.8
42.1	43.6

**Table 2:** Doses employed on the *Salmonella typhimurium* Ames test.

*Citrus sinensis* (ng/petri dish)	*Citrus latifolia* (ng/petri dish)
0.842	0.872
0.0842	0.0872
0.00842	0.00872

**Table 3:** Chemical compositions of *Citrus sinensi*s and *Citrus latifolia* EOs by GC-MS analysis.

RETENTION TIME	COMPOUND	ABUNDANCE (%)
**COMPOSITION OF** ***C. sinensis*** **ESSENTIAL OIL**		
5.61	α-pinene	0.799
6.36	β -pinene	0.358
6.68	α -myrcene	2.796
7.4	d-limonene	96.046
**COMPOSITION OF** ***C. latifolia*** **ESSENTIAL OIL**		
5.49	α-thujene	0.48
5.61	α-pinene	2.17
6.35	β-thujene	14.85
6.4	β -pinene	12.79
6.69	α -myrcene	1.43
7.39	d-limonene	51.64
7.99	γ-terpinene	12.80
11.41	Citral B	0.56
11.95	*3,7-Nonadien-2-one, 8-methyl-, (E)*-	0.93
13.52	geranyl propionate	0.99
14.5	β -farnesene	0.40
14.71	α -bergamote	0.96
15.87	β-bisabolene	1.25*10^−5^

Retention times were measured in minutes. Abundance percentage was calculated as a ratio of the area under the curve of each peak and the total area.

**Table 4:** Inhibitory Concentration at 50% of growth (IC_50_) and Minimum Inhibitory Concentrations (MIC).

	*C. sinensis*		*C. latifolia*	
	IC_50_	MIC	IC_50_	MIC
*C. albicans*	8.41	1.68	4.18	0.40
*C. tropicalis*	5.50	0.72	7.54	1.3
*C. glabrata*	3.82	0.42	3.11	0.22
*C. lusitaniae*	12.50	3.71	6.90	1.09
*C. guilliermondii*	–	–	2.92	0.19

All concentrations are expressed as total μg added. Dashed lines indicate incalculable values.
